# Potential of Moroccan entomopathogenic nematodes for the control of the Mediterranean fruit fly *Ceratitis capitata* Wiedemann (Diptera: Tephritidae)

**DOI:** 10.1038/s41598-020-76170-7

**Published:** 2020-11-05

**Authors:** Fouad Mokrini, Salah-Eddine Laasli, Youssef Benseddik, Abdelmalek Boutaleb Joutei, Abdelali Blenzar, Hicham Lakhal, Mohamed Sbaghi, Mustafa Imren, Göksel Özer, Timothy Paulitz, Rachid Lahlali, Abdelfattah A. Dababat

**Affiliations:** 1 Biotechnology Unit, Regional Center of Agricultural Research, INRA-Morocco, Rabat, Morocco; 2 Laboratory of Botany, Mycology and Environment, Faculty of Science, Mohammed V University, Rabat, Morocco; 3 Department of Biology, Meknès, Faculty of Sciences, Moulay Ismail University, PO Box 11201, Zitoune, Meknès, Morocco; 4 Department of Plant and Environment Protection, Ecole Nationale D’Agriculture de Meknes, km. 10, Route Haj Kaddour, B.P. S/40, 50001 Meknès, Morocco; 5 Head Quality and Postharvest Citrus Department, Delassus Group, Casablanca, Morocco; 6 Department of Plant Protection, Faculty of Agriculture and Natural Sciences, Bolu Abant Izzet Baysal University, Bolu, 14030 Turkey; 7 United, States Department of Agriculture, Agricultural Research Service, Wheat Health, Genetics and Quality Research Unit, Washington State University, Pullman, WA 99164-6430 USA; 8 International Maize and Wheat Improvement Center (CIMMYT), P.K. 39, Emek, Ankara, 06511 Turkey

**Keywords:** Plant sciences, Pathogens

## Abstract

The Mediterranean fruit fly, *Ceratitis capitata* Wiedemann, is a deleterious pest worldwide affecting fruit production. The entomopathogenic nematodes (EPNs) are a potential biocontrol agent that could be effectively used to control this Mediterranean fruit fly. In this study, five EPN strains reported from different fields in Morocco were evaluated for their efficacy against *C. capitata*. In laboratory assays, *Steinernema feltiae*-SF-MOR9, *S. feltiae*-SF-MOR10 and *Heterorhabditis bacteriophora*-HB-MOR7 strains showed significantly higher infectivity and penetration rates when compared to the other strains. *S. feltiae*-SF-MOR9 caused the highest larval mortality rate (80%) at 50 infective juveniles (IJs) cm^−2^. However, additional results showed that both *S. feltiae* strains were significantly effective in controlling *C. capitata* larvae in apricot (*Prunus armeniaca*) fruits on soil surface with high mortality rate at 50 and 100 IJs cm^−2^. Different soil textures and moisture levels resulted in a significant variation in EPN strain virulence against *C. capitata*. Sandy clay loam soil in combination with 50 IJs cm^−2^ of *S. feltiae* (SF-MOR9 or SF-MOR10*)* caused a higher mortality rate of *C. capitata* larvae. Furthermore, applying these EPN strains at 50–100 IJs cm^−2^ in combination with 10–15% moisture level showed optimal results against *C. capitata* larvae. Therefore, those two Moroccan EPN strains could be used as promising eco-friendly biological agents against *C. capitata*.

## Introduction

The Mediterranean fruit fly (Medfly), *Ceratitis capitata* Wiedemann, is one of the most economically important tephritid fruit fly pests that cause loss of infested fruits^1^. Medfly females deposit eggs into fruits, thereby, making infested fruits not suitable for consumption or marketing. The economic damage caused by Medfly to fruits has been estimated to be at least 2 billion dollars annually worldwide^2^. In Morocco, Medfly infests fruits of citrus (*Citrus sinensis* and *Citrus reticulata*), pears (*Pyrus communis*), apples (*Malus domestica*), apricots (*Prunus armeniaca*), and peaches (*Prunus persica*)^3^. In addition to the direct losses of infested fruits, indirect losses due to quarantine restrictions imposed on fresh fruit from Medfly-infested countries create serious limitations in terms of export^4,5^.

Insecticide applications have been extensively used to control Medfly^6,7^. However, their potential adverse effects including the toxicity to non-target organisms, insect resistance to insecticides, environmental pollution and residues on food demand the investigation of alternative biological control measures^8^. In this context, the entomopathogenic nematodes (EPNs) of the genera *Steinernema* (Panagrolaimomorpha: Steinernematidae) and *Heterorhabditis* (Rhabditomorpha: Heterorhabditidae) are successfully used to control a great variety of soil-borne insect pests around the world^9–11^. Once the infective juveniles (IJs), which is the only free-living stage of nematodes, enter hosts primarily via body openings such as anus, spiracles or mouth^12,13^, symbiotic bacteria (*Xenorhabdus* and *Photorhabdus* in *Steinernema* and *Heterorhabditis*, respectively) are released from their intestines into the hemocoel of target insects^14,15^, which results in insect death within 24–48 h^14^. Native EPN strains/species are more adapted to local climatic conditions and are, therefore, more likely to survive in the target area after their application to soil^15^. Several studies have shown the potential of indigenous EPN as good biocontrol agents against a broad range of soil insect pests including the dipteran (or tephritid) species pests, due to their adaptations to local environmental conditions^17–20^. In our recent surveys for EPNs, we identified several strains of *Steinernema feltiae* and *Heterorhabditis bacteriophora* that are native to Morocco^16,21^. Nonetheless, pathogenicity of strains of EPN species to arthropod pests often varies between EPN strains, suggesting the need to assess and measure the infectivity traits of the Moroccan EPN strains against the Medfly. In effect, this will provide sufficient evidence to select the more promising EPN strains for the control of Medfly in orchards. To the best of our knowledge, the efficiency of these native EPN strains, isolated from soils in Morocco had not been evaluated against *C. capitata*. Thus, we investigated the pathogenicity of five native EPN strains against the third-instar larvae and pupae of *C. capitata* using a series of laboratory and glasshouse trials. Therefore, the main objective of this study was to select the most effective EPN strains to control *C. capitata* pest in Morocco under different soil types and soil moisture contents.

## Results

### Laboratory trial: Pathogenicity of five native entomopathogenic nematode species strains to *C. capitata* larvae

**Figure 1:**
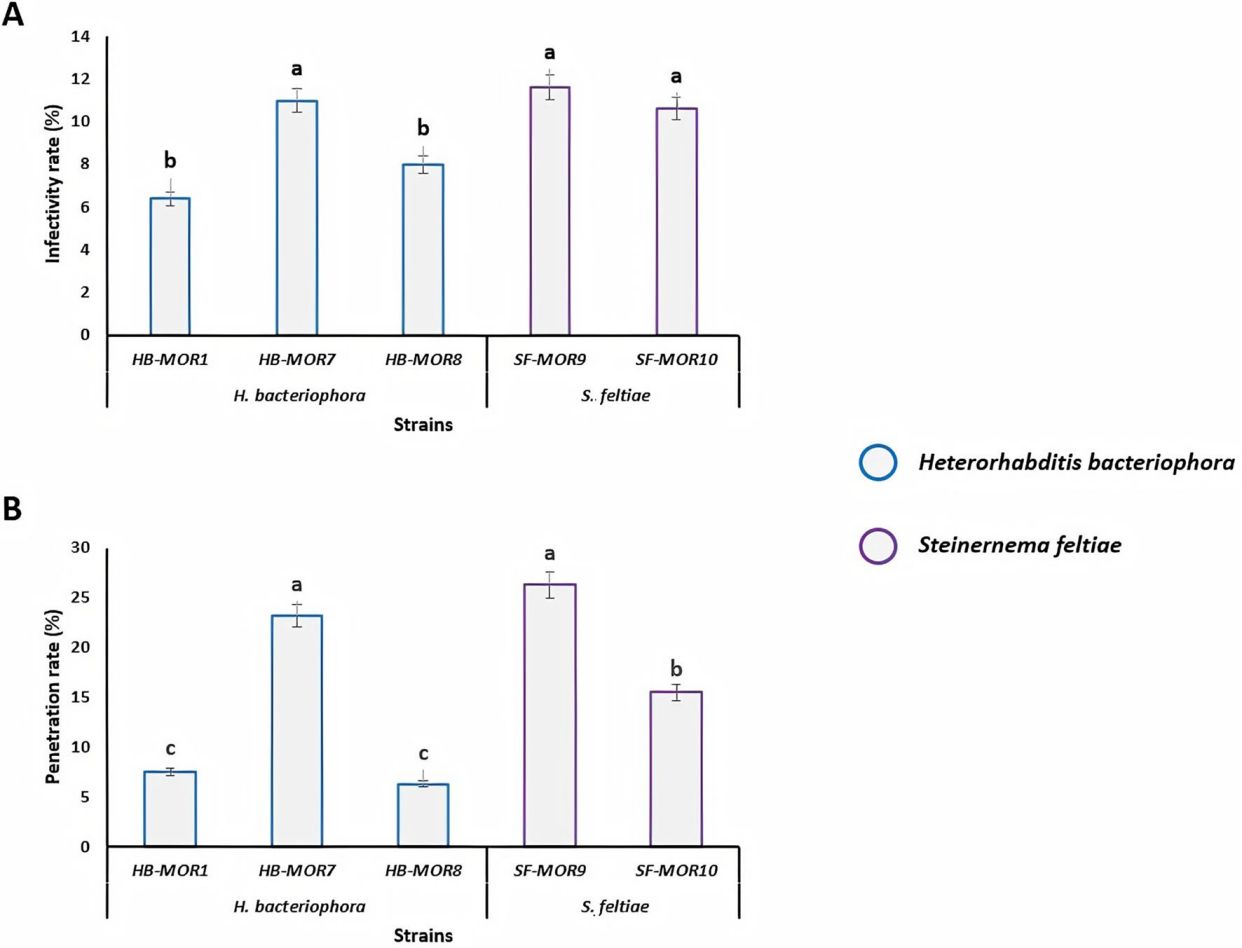
Pathogenicity of Moroccan EPN strains of *Steinernema feltiae* (SF-MOR9 and SF-MOR10) and *H. bacteriophora* (HB-MOR1, HB-MOR7 and HB-MOR10) against *C. capitata* (laboratory trial). (**A**) Larval infectivity rates. (**B**) Penetration rates. Letters represent homogeneous groups based on protected least significant difference test (LSD) for each variable at (*P* < 0.001). Error lines on the bars represent the standard error.

Moroccan EPN strains of *H. bacteriophora* (HB-MOR1, HB-MOR7 and HB-MOR8) and *S. feltiae* (SF-MOR9 and SF-MOR10) were assessed in terms of their pathogenicity against *C. capitate* based on their infectivity and penetration rates. The results of laboratory trial showed that *H. bacteriophora*-MOR7 had a significantly higher infectivity rate (*F*_*index*_ = 38.64; *df* = 4; *P* < 0.001) compared to the other strains of the same species (Fig. 1A). Both *Steinernema* strains (SF-MOR9 and SF-MOR10) showed high infectivity although no significant difference was observed between the strains (*F*_*index*_ = 23.18; *df* = 4; *P* = 0.09). As for the penetration rate of nematodes into *C. capitata* larvae, the same trend of efficacy was observed for *Heterorhabditis* (HB-MOR7) and *Steinernema* (SF-MOR9) strains (*F*_*index*_ = 44.1; *df* = 4; *P* < 0.001). Unlike infectivity rate, *S. feltiae*-MOR10 strain differed significantly from SF-MOR9 with a low penetration rate (Fig. 1B).

### Glasshouse trial 1: Entomopathogenic nematode-treatments against *C. capitata*-infested apricot fruit

The susceptibility of the third-instar larvae to five native EPN strains was investigated inside apricot fruits. The first glasshouse trial showed mortality rates caused by all tested EPN strains (*F*_*index*_ = 13.8; *df* = 4; *P* < 0.001) (Table 1). *Steinernema feltiae-*MOR9 strain provided the highest mortality rates (80 and 77%) at 50 and 100 IJs cm^−2^, respectively, while the lowest rates of 30.9 and 23.7% were observed at 50 IJs cm^−2^ of *H. bacteriophora*-MOR1 and 100 IJs cm^−2^ of *H. bacteriophora*-MOR8, respectively. Moreover, infected larvae were differently distributed either inside fruits or in the soil. Most of the larvae controlled by *S. feltiae* strains at different concentrations were significantly found inside apricot fruits (*P* < 0.001). However, *H. bacteriophora* strains induced higher virulence levels to the larval stage in the soil.

**Table 1:** Overall larval mortality of *C. capitata* caused by different EPN strains at 50 and 100 IJs cm^−2^ and percentage that died in 6 infested apricots compared to those in the soil (Glasshouse trial 1).

Nematode species	Strains	Concentration (IJs/cm^2^)	Total larvae	Infected larvae of *C. capitata* ± SEM^b^		Percent^a^ overall larval mortality ± SEM^b^
				Inside apricot fruits^a^	Soil^a^	
*Heterorhabditis bacteriophora*	HB-MOR1	50	55	5 ± 2cd	12 ± 2ab	30.9 ± 3.2bcd
		100	54	7 ± 3.1c	18 ± 3a	46.2 ± 1.3bc
	HB-MOR7	50	49	9 ± 5.3c	20 ± 2a	58 ± 4.1b
		100	51	8 ± 2c	16 ± 4a	47 ± 3.7bc
	HB-MOR8	50	50	6 ± 2cd	13 ± 3ab	38 ± 2.3bcd
		100	59	5 ± 1cd	9 ± 2b	23.7 ± 2.9d
*Steinernema feltiae*	SF-MOR9	50	50	29 ± 3a	11 ± 3ab	80 ± 5.7a
		100	53	31 ± 4a	10 ± 3ab	77.3 ± 4.8a
	SF-MOR10	50	48	22 ± 3 ab	9 ± 2b	64 ± 3.1ab
		100	53	20 ± 2 ab	8 ± 1b	52 ± 5.2b

^a^ Values followed by the same letter are not significantly different (LSD test, *P* < 0.05).

^b^ SEM values represent the Standard Error of the Mean.

### Glasshouse trial 2: The effect of density of entomopathogenic nematode strains on *C. capitata* mortality

**Figure 2:**
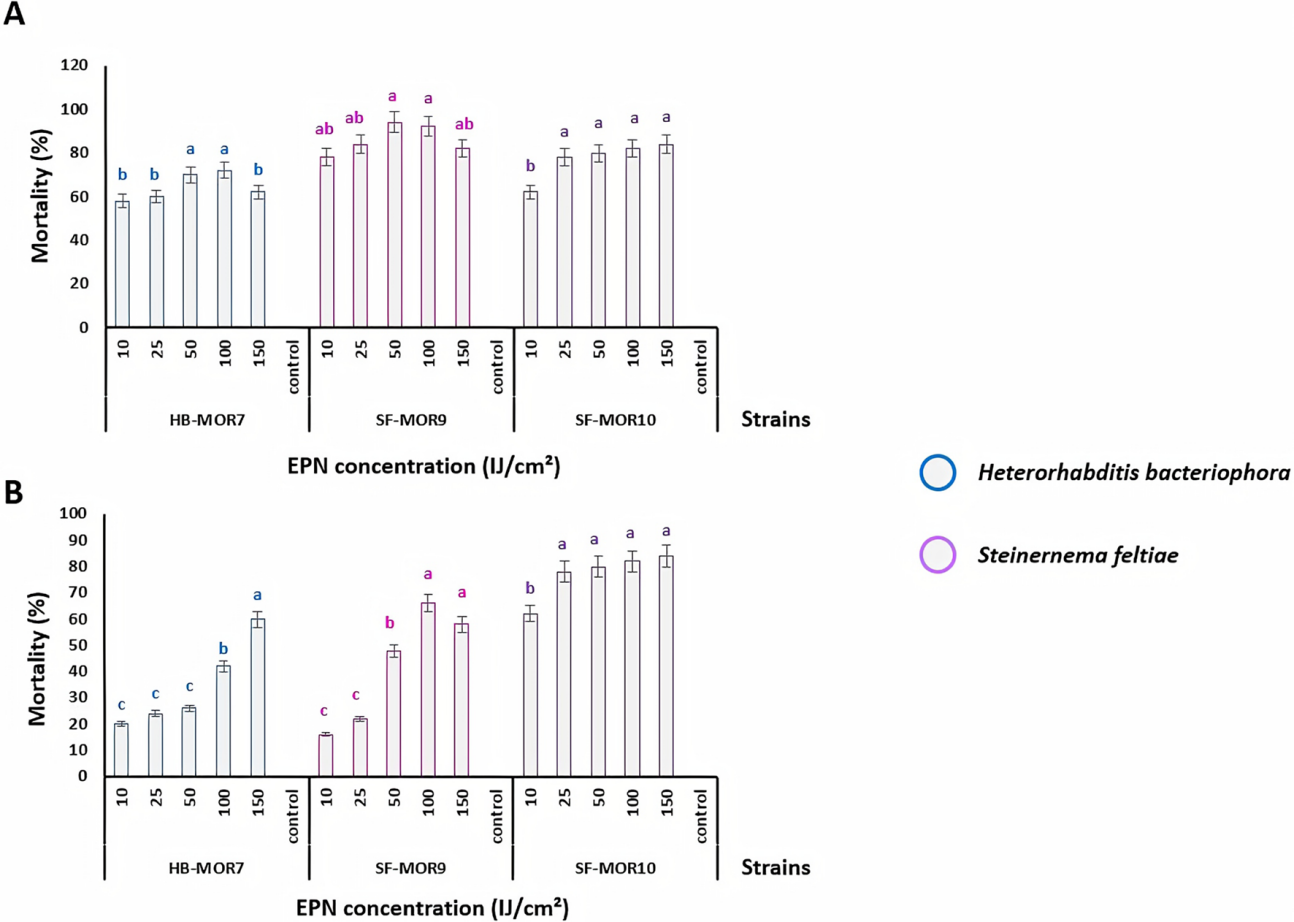
Effect of applied EPN (*S. feltiae* and *H. bacteriophora*) concentration against *Ceratitis capitata* susceptible life stages (Glasshouse trial 2). (**A**) Larval mortality rates under five concentrations (10, 25, 50, 100, and 150 IJs cm^−2^), control was without any EPNs. (**B**) Pupal mortality rates. Letters represent homogeneous groups based on protected least significant difference test (LSD) for each variable at (*P* < 0.001). Error lines on the bars represent the standard error.

A glasshouse trial was conducted to accurately assess the impact of EPN strain concentrations (10, 25, 50, 100, and 150 IJs cm^−2^) against *C. capitata* larvae and pupae. Three (HB-MOR7, SF-MOR9, and SF-MOR10) out of the five EPN tested strains were selected due to their higher infectivity and penetration rates. Regardless of strains, two concentrations (50 and 100 IJs cm^−2^) exhibited significant mortality rates (up to 94 and 92%, respectively) against *C. capitata* larvae (Fig. 2A), (*F*_*index*_= 18.91; *df* = 4; *P* < 0.001). Moreover, *Steinernema* strains were more effective against the larval stage of *C. capitata* with a significant higher mortality rate (*F*_*index*_ = 38.66; *df* = 2; *P* < 0.001). This mortality was completely different from that obtained for the pupal stage of *C. capitata* (Fig. 2B). The virulence of HB-MOR7 significantly increased *C. capitata* mortality rate to 60% (*F*_*index*_ = 10.42; *df* = 4;* P* < 0.001) at the concentration of 150 IJs cm^−2^. With *Steinernema* strains, the highest efficacy on pupae was generally obtained at the concentrations (50 and 100 IJs cm^−2^) with mortality of 85% (*F*_*index*_ = 33.5; *df* = 4; *P* < 0.001). On the other hand, SF-MOR10 showed an opposite trend as the highest mortality rate (78%) was obtained at the lowest concentration (25 IJs cm^−2^), which indicates the effectiveness of this particular strain against larval and pupal stages of *C. capitata*.

**Figure 3:**
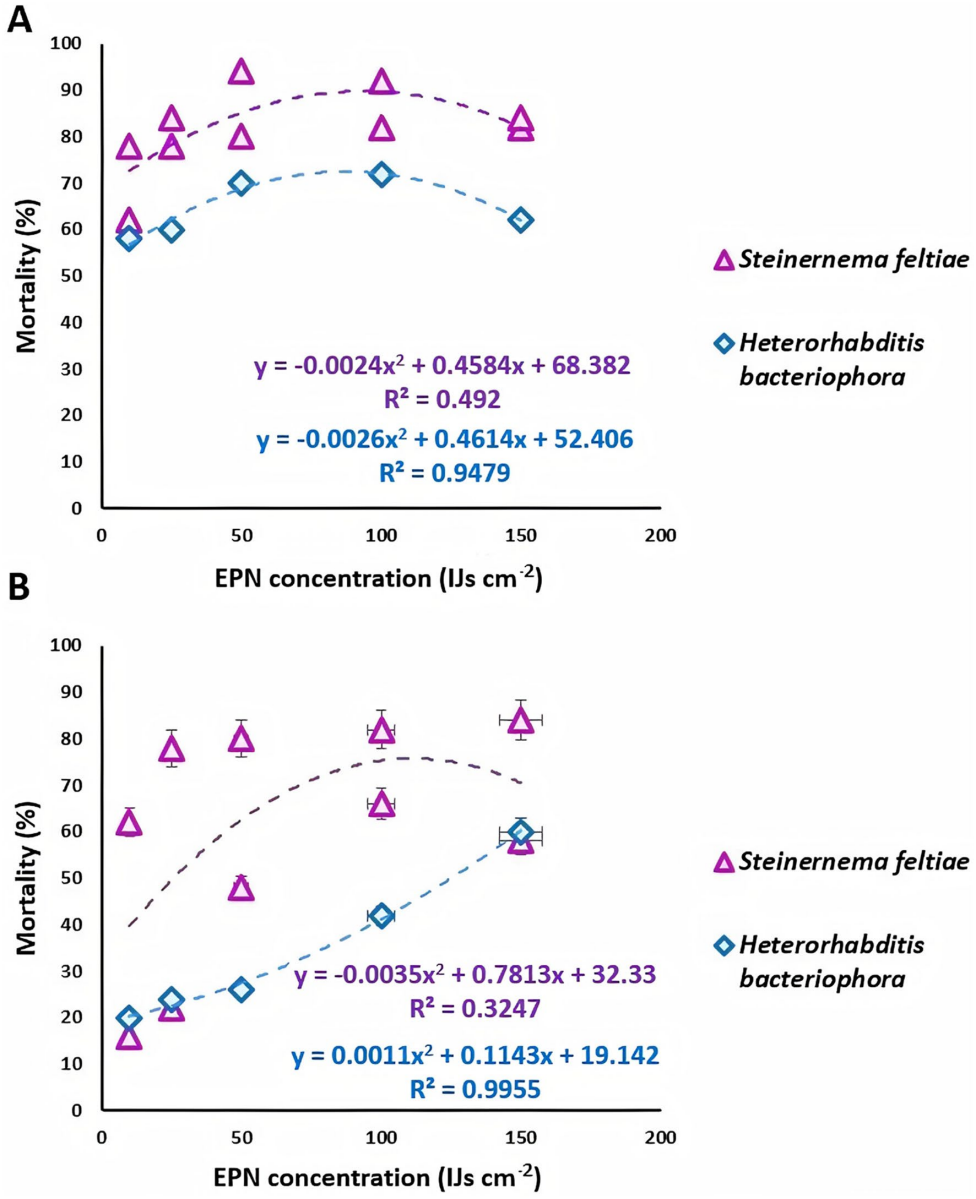
Polynomial regression analysis for the relationship between *C. capitata* mortality and EPN (*S. feltiae* and *H. bacteriophora*) concentrations applied. (**A**) Larval stage. (**B**) Pupal stage. All values of R^2^ were significant at *P* < 0.001. Error lines represent the standard error.

To confirm the relationship between insect mortality and EPN concentration, polynomial regression analyses were performed (Fig. 3). For the larval stage, significant regression was obtained for both EPN species studied (*P* = 0.0007). However, *H. bacteriophora* (HB-MOR7) strain was highly related to the larval mortality (*R*^2^ = 0.947) compared to *S. feltiae* strain (*R*^2^ = 0.492) (Fig. 3A). The same correlation was observed for the pupal stage as well (Fig. 3B). The relationship between EPN concentration and mortality was even greater for *H. bacteriophora* (*R*^2^ = 0.99) while it was a bit lower for *Steinernema* (*R*^2^ = 0.324) probably due to the difference observed in concentration patterns.

### Glasshouse trial 3: Effect of soil texture and moisture levels on the virulence of entomopathogenic nematodes to *C. capitata*

**Figure 4:**
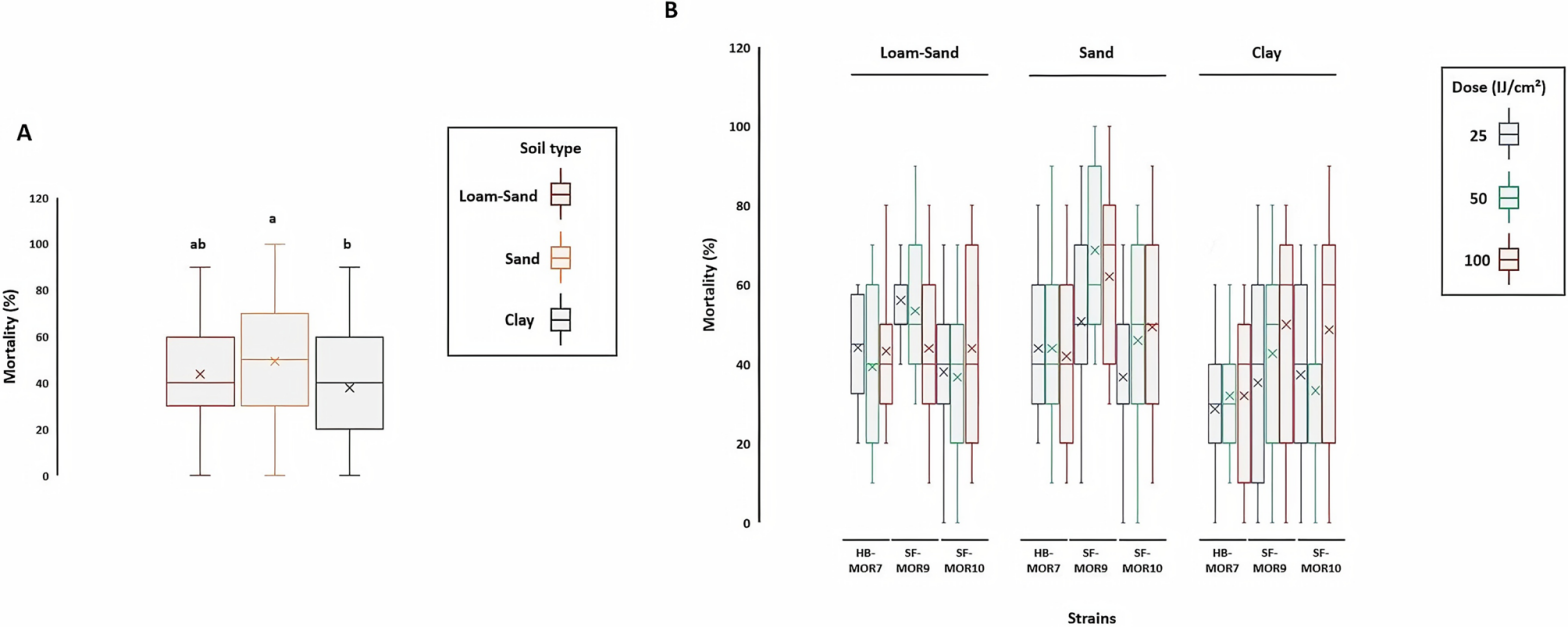
Soil texture impact on Moroccan EPN strain virulence against *C. capitata* larval stage (Glasshouse trial 3). (**A**) Box and whisker plot showing the average mortality rate of insect larval stage using three types of soil (loam-sand, sandy clay loam, and clay). (**B**) Box and whisker plot showing the interaction effect of soil texture and EPN concentration (IJs cm^−2^) on larval mortality. Letters represent homogeneous groups based on protected least significant difference test (LSD) for each variable at (*P* < 0.001). Middle boxplot line represents the median (Q2) while the (x) spots represents the mean. Quartiles Q1 and Q3 were calculated accordingly for each treatment. Error lines represent the maximum and minimum values, respectively. Figure prepared in R (version 3.4.3, R Core Team^48^, https://www.r-project.org/), using ggplot2^50^.

The effect of soil type in combination with moisture level under different EPN concentrations was investigated. Three soil types were applied: loamy sand, sandy clay loam, and clay with three EPNs concentrations (25, 50, and 100 IJs cm^−2^). In most cases, there were significant differences between soil types on the mortality of *C. capitata* larval stage (Fig. 4A). Sandy clay loam soil was significantly efficient to enhance the destruction of insect larvae with more than 50% mortality rate (*F*_*index*_ = 39.63; *df* = 2; *P* < 0.001). Loamy sand soil also showed a prominent efficacy (< 50% mortality), indicating the importance of soil texture in the control of Medflies. The results of this study indicated that there is a significant interaction between soil and EPN concentration (*F*_*index*_ = 5.63; *df* = 4; *P* < 0.001). The larval mortality was significantly optimal at a 50 IJs cm^−2^ of *S. feltiae* strain (SF-MOR9) in sandy clay loam soil with 70% mortality (Fig. 4B). In contrast, the combination of clay soil with low EPN concentration (25 IJs cm^−2^) allowed the lowest larval mortality rate (25%).

**Figure 5:**
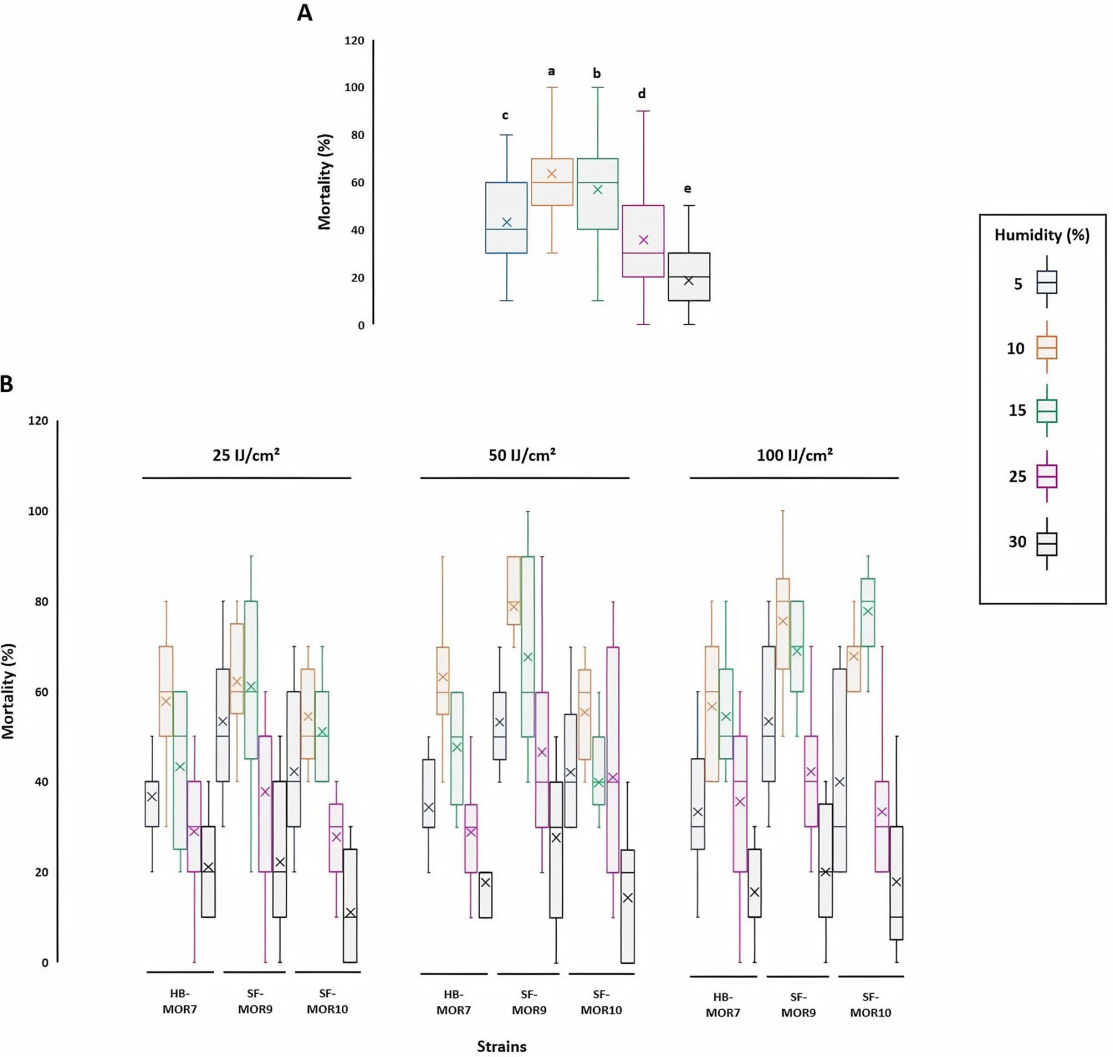
Moisture content soil humidity effect on Moroccan EPN strains virulence against *C. capitata* larval stage (Glasshouse trial 3). (**A**) Box and whisker plot showing the average mortality rate of insect larval stage using five moisture content (5, 10, 15, 25 and 30%). (**B**) Box and whisker plot showing the interaction effect of moisture content and EPN concentration (IJs cm^−2^) on larval mortality. Letters represent homogeneous groups based on protected least significant difference test (LSD) for each variable at (*P* < 0.001). Middle boxplot line represents the median (Q2) while the (x) spots represents the mean. Quartiles Q1 and Q3 were calculated accordingly for each treatment. Error lines represent the maximum and minimum values, respectively. Figure prepared in R (version 3.4.3, R Core Team^48^, https://www.r-project.org/), using ggplot2^50^.

To assess the effect of moisture levels on the larval mortality, five moisture levels (5, 10, 15, 25, and 30%) were applied alongside with 3 EPN concentrations (25, 50, and 100 IJs cm^−2^) of the most virulent strains (HB-MOR7, SF-MOR9, and SF-MOR10). Results indicated that 10% moisture content could increase significantly the insect mortality rate (> 60%) (*F*_*index*_ = 227.10; *df* = 4; *P* < 0.001) (Fig. 5A). Alternatively, this humidity positively interacted with EPN strains used (*F*_*index*_ = 2.187; *df* = 8; *P* = 0.029) and the applied concentration (*P* < 0.001). Optimal result (> 80% larval mortality) was recorded by using SF-MOR9 strain at 50 IJs cm^−2^ combined with 10% moisture content (Fig. 5B). The same pattern was observed when the applied EPN concentration increased to 100 IJs cm^−2^ with SF-MOR9 and SF-MOR10 and either 10 or 15% moisture contents, respectively. *Heterorhabditis bacteriophora* strain (HB-MOR7) had significantly lower mortality rates (< 50%). However, better efficacy was recorded at 25 and 50 IJs cm^−2^ concentrations and 10% moisture content.

## Discussion

In the present study, the pathogenicity of five native EPN strains collected from Morocco was investigated against *C. capitata*, a serious threat for the Moroccan fruit production. Laboratory screening experiments of *C. capitata* mortality induced by different EPN strains were then required before conducting field trials for reducing the number of strains. Our results indicated that the third-instar larvae of *C. capitata* are moderately to highly susceptible to 5 EPN strains. This confirms reports of several previous studies highlighting the efficacy of EPN as natural enemies to *C. capitata*^22,23^. The highest larval mortality rates were obtained with *S. feltiae*-MOR9 strain (96%), followed respectively by *H. bacteriophora-*MOR7 and *S. feltiae*-MOR10 strains with 90 and 83% mortality rates. The differences in the mortality rates among EPN strains/species could be attributed to the foraging strategy of IJs, as well as the behavior of *C. capitata* third-instar larvae. *Steinernema feltiae* have an intermediate forage strategy^13^, whereas *H. bacteriophora* displays a cruise strategy^24^. By considering the effectiveness of the three EPN strains, our results are in complete agreement with those of James et al.^20^, who reported more than 90% larval mortality of *C. capitata* caused by South African *Heterorhabditis indica* and *H. noenieputensis* strains. In a similar study, Sirjani et al*.*^18^ reported higher pathogenicity of *S. feltiae* than *H. bacteriophora* against the olive fruit fly larvae, *Bactrocera oleae*.

In the apricot assay, the ability of EPN strains to enter apricots and infect the larvae both inside and when they left the fruits were examined. Results from our study showed that IJs were able to penetrate and infect larvae before they exited the fruit. The highest overall larval mortality rates caused by *S. feltiae*-MOR9 strain were 80 and 77% at 50 and 100 IJs cm^2^, respectively. In addition, IJs of *S. feltiae*-MOR9 strain were more infectious against larvae inside apricots than in the soil, suggesting that their penetration in the fruit was via any tear in the fruit skin or through oviposition punctures caused by *C. capitata* females^25^. Moreover, Hübner et al*.*^26^ suggested that IJs of *S. feltiae* were capable of locating oviposition punctures on the fruit surface and they could accumulate faster in the fruit. Several studies reported the susceptibility of several dipteran larvae inside fruit to EPN^18,25,26^. Sirjani et al*.*^18^ demonstrated that IJs of *S. feltiae* entered the fallen olives and they were able to infect *Bactrocera oleae* Rossi (Diptera: Tephritidae) larvae. However, Hübner et al*.*^26^ underlined that the number of living IJs of *S. feltiae* inside the fruit were reduced due to its higher sensitivity to fruit acidity. This confirms our results, demonstrating the decreased infectivity of all *H. bacteriophora* strains only inside apricots.

Several reports have investigated the IJs concentration of EPN for controlling several tephritid fruit fly species^27–29^. However, the adequate concentration may vary with regards to EPN strain/species, target insect pests, and other environmental factors, such as soil textures and moisture levels^30^. The results regarding the effect of EPN concentrations emphasized that the highest levels of *C. capitata* larval mortality rates (82 and 94%) were provided by both strains, *S. feltiae*-MOR10 and *S. feltiae*-MOR9, at both 100 and 50 IJs cm^−2^, respectively. Interestingly, 50 IJs cm^−2^ was significant enough to cause more than 80% larval mortality of *C. capitata*, suggesting that EPN concentrations higher than 100 IJs cm^−2^ may cause intraspecific competition among IJs of the same EPN species or strain. This nematode concentration is lower than reported in previous studies^22,31,33^. Gazit et al.^22^ reported that the highest larval mortality (82.5%) of *C. capitata* was achieved when *S. riobrave* was applied at a concentration of 100 IJs cm^−2^ compared to other concentrations. Minas et al*.*^32^ pointed out that *H. baujardi* strain provided good control of *C. capitata* larvae at the highest concentration (273 IJs cm^−2^). A 100 IJs cm^−2^ concentration of *S. feltiae* caused 78% of larval mortality of *C. capitata*^31^, while in our study, 94 and 82% mortality rates were achieved when *S. feltiae*-MOR9 and *S. feltiae*-MOR10 were applied at 50 and 100 IJs cm^−2^, respectively. These differences in IJs concentrations may be attributed to variations in experimental methods^33^. When selected EPN isolates were tested against pupae of *C. capitata*, the mortality rates were generally low regardless of IJs concentration differences compared to *C. capitata* larvae. Although larvae of *C. capitata* generally spend a few days (2–5) in the fruit-to-soil before pupation, the infectivity of the studied EPN strains was higher to larvae than pupae of *C. capitata*. These results confirm the earlier finding of Kepenekci and Susurluk^34^ who demonstrated that Turkish strains of *S. feltiae* caused the lowest mortality (26.6–40%) of *C. capitata* pupae at 50 and 100 IJs/insect. Our findings suggest that both strains, *S. feltiae*-MOR10 and *S. feltiae*-MOR9 may be able to find and infect the third-instar larvae and pupae of *C. capitata* in the soil.

The soil type and moisture level can affect EPN pathogenicity against *C. capitata*^35^, understanding their effects is crucial to determine appropriate management strategy. But effects can vary with EPN strains/species^36^. In this study, soil texture and moisture level had a significant impact on the overall mortality of *C. capitata* exposed to different EPN strains. Our results showed that sandy clay loam soil at low moistures (10 and 15%) may favor nematode movement compared to other soil types/moisture levels. At a concentration of 50 IJs cm^−2^, *S. feltiae*-MOR9 strain caused the highest mortalities (93.3 and 90%) in sandy clay loam soil at 15 and 10% moisture levels, respectively. The highest efficacy of *H. bacteriophora*-HB-MOR7 strain was achieved in sandy clay loam soil with a moisture level of 15%, leading to a mortality rate of up to 80%. This effectiveness could be explained by a decrease in water content between soil particles or expansion of soil pore space, facilitating nematode migration^37^. However, low mortality in control was observed in clay soil with a moisture level of 30%. This low efficiency of the tested EPNs is more likely related to an insufficient supply of oxygen to *C. capitata* due to soil saturation. Our results confirmed previous studies on the efficacy of *S. feltiae* and *H. bacteriophora* against other tephritid fruit fly species under many environmental factors, such as soil texture^38,39^ and soil moisture^33,40^. Rohde et al*.*^41^ reported that *H. bacteriophora* HP88 infectivity against late third-instars of *C. capitata* increased in sandy soil at 10% moisture level, compared to S*. riobrave* ML29. Langford et al.^40^ indicated that *S. feltiae* caused high mortality against Queensland fruit fly, *Bactrocera tryoni* at different concentrations, and over a wide moisture range than both *S. carpocapsae* and *H. bacteriophora* which caused high efficacy but was more limited to the high IJ concentration and lower moisture.

Overall, our results highlighted the potential of both strains of *S. feltiae*-MOR9 and *S. feltiae*-MOR10 as biocontrol agents against third-instar *C. capitata* larvae, which arise from fruit and complete their pupation in the ground. EPNs at this stage can significantly reduce the number of pupae and adults in Moroccan fruit orchards, mainly grown in sandy clay loam soil. Since EPNs are also able to enter dropped fruits and infect larvae of *C. capitata* inside these fruits, their use as soil drench treatments could be an appropriate fruit fly control measure, notably when many infested fruits fall on the ground, as is often the case of citrus and apricot orchards with a longer harvesting period. Nevertheless, additional field experiments are still required to better evaluate the effectiveness of native EPN strains under field conditions.

## Methods

### *Ceratitis capitata* colonies

*Ceratitis capitata* larvae were retrieved from naturally-infested apricot fruits collected from infested apricot orchards in northern Morocco. The larvae were reared in sterilized sand (moisture content = 10%) using cages (41 × 34 × 50 cm^3^) which were incubated for one week at a temperature of 25 ± 2 °C, 70 ± 10% of RH, and 12-h photoperiod until they developed to pupae. The pupae were used to establish Medfly colonies. The third instar larvae and pupae of *C. capitata* obtained from the colonies were used for laboratory and glasshouse trials.

### Native entomopathogenic nematodes for the trials

Three *Heterorhabditis bacteriophora* and two *Steinernema feltiae* (Table 2), recently retrieved from soil in Morocco^21^, were used to infect susceptible late instar larvae of *Galleria mellonella* (Lepidoptera: Phyralidae). The EPN-killed *G. mellonella* larvae were transferred to White traps and then incubated at 25 °C until active infective juveniles (IJs) emerged^42,43^ and daily collected from the White traps for 7 consecutive days and then were stored at 10 °C in a 0.5-L container filled with distilled water. To check their activity, IJs containers were brought to room temperatures for 1 h and the viability of active IJ stock suspensions was checked by observing movement of IJs under a stereomicroscope^44^. Only active IJs were used in all trials.

**Table 2:** The five studied EPN strains from Morocco.

Nematode species	Isolate	Geographical coordinates in Morocco	GenBank accession no	Vegetation
*Heterorhabditis bacteriophora*	HB-MOR1	33° 49′ 00.2″ N 05° 30′ 40.2″ W	MN420270	Potato (*Solanum tuberosum* L.)
	HB-MOR7	33° 49′ 03.2″ N 05° 19′ 25.9″ W	MN420696	Plum trees (*Prunus* sp.)
	HB-MOR8	33° 59′ 13.7″ N 05° 29′ 16.3″ W	MN420697	Olive trees (*Olea europaea* L.)
*Steinernema feltiae*	SF-MOR9	31° 29′ 46.5″ N 04° 24′ 32.7″ W	MN749619	Date palms (*Phoenix dactylifera* L.)
	SF-MOR10	33° 40′ 03.3″ N 05° 02′ 19.5″ W	MN752176	Oak forest (*Quercus* L.)

### Laboratory trial: Pathogenicity of five native entomopathogenic nematode species strains to third instar *C. capitata* larvae

A 12-well-half-plate of a 24-well plate (Greiner Bio-One CELLSTAR, Vilvorde, Belgium; 3.14 cm^2^ surface area/ well) was used to evaluate pathogenicity of each EPN species strain to third-instar larvae. The bottom of each alternate well was lined with a circular filter paper (diameter = 13 mm). Each alternate bioassay well with a single 3rd instar *C. capitata* larvae was inoculated with 100 IJs/50 μl of tap water for each tested species of EPN. Plates were organized in a completely randomized block design with five replications (each replication was represented by one 12-well plate for each EPN treatment or the control). Treatments which served as controls were treated with tap water only. All plates were covered with a lid and then incubated for 2 days at 25 ± 2 °C. Dead larvae were assessed and transferred to Petri dishes (diameter = 9 cm) lined with filter paper. The number of IJs penetrated each larva was counted by dissecting and observing each cadaver under a stereomicroscope. The experiments were independently repeated for a second time to validate the results.

### Glasshouse trial 1: Entomopathogenic nematode-treatments applied to *C. capitata*-infested apricot fruit

About 1500 g of sterilized sand with 10% moisture content was added into plastic containers (34.5 × 22.5 × 19 cm^3^). Each container was infested with six naturally-infested apricot fruits, which contained 9–13 *C. capitata* larvae based on our initial assessment, by adding them to the soil surface. Nematodes were applied at the rates of 50 and 100 IJs cm^−2^ over the apricot fruits using a hand-held water sprayer. Plastic containers were arranged in a completely randomized block design with five replications. Controls were treated with distilled water only. The experimental units were covered with a transparent plastic sheet and then incubated for four days at 25 ± 2 °C. The apricot fruits were desiccated, and soil in each container was sieved to retrieve larvae and pupae, which were checked for EPN infections under a microscope. The experiment was independently repeated for data validation.

### Glasshouse trial 2: The effect of density of entomopathogenic nematode strains on *C. capitata* mortality

The three most virulent strains, *H. bacteriophora* HB-MOR7, *S. feltiae* SF-MOR9, and *S. feltiae* SF-MOR10 (Table 2), identified in laboratory trial and glasshouse trial 1 were selected to study the effect of different IJs concentrations on the third-instar larvae as well as on pupae of *C. capitata*. Sand assay in plastic pots (6.5-cm diameter, and 33.1 cm^2^ surface area) was used for this trial. Each plastic pot was filled with 30 g of sterilized sand with 10% moisture content. Each pot received 10, 25, 50, 100 or 150 IJs cm^−2^. Each treatment was replicated 5 times placed in a randomized block design. Twenty-four hours after EPN was applied to sand in the pots, ten pupae or 10 third-instar larvae from the reared Medfly colonies were added to soil surface per each pot. Controls were treated with 1 ml of sterilized distilled water. All pots were covered with Parafilm and then incubated for 2 weeks at 25 ± 2 °C. Larval mortality caused by EPN was assessed after confirming IJs in dead larvae under a microscope, similar to the procedures used in laboratory trial 1 and glasshouse trial 1. However, pupal mortality was determined by subtracting the number of emerging adults from the initial number of pupae added to EPN-treatment^22^. The experiment was repeated twice for data validation.

### Glasshouse trial 3: Effect of soil texture and moisture contents on virulence of entomopathogenic nematode strains to *C. capitata*

The effect of the selected EPN strains against pupae and third-instar larvae of *C. capitata* under different soil types and at different moisture levels was investigated in a completely randomized design with five replicates. Three types of soil, viz*.* loamy sand (80% sand, 5% silt, 15% clay, and pH 7.5), sandy clay loam (45% sand, 21% silt, 34% clay, and pH 7.3), and clay (30% sand, 30% silt, 40% clay, and pH 8.5) were collected from citrus orchards in northern Morocco. Experiments were performed in plastic pots (5 cm base diameter, 8 cm height, and 7 cm top diameter). Each pot was filled with 90 g of each sterilized soil type. Distilled water was added to soil in pots until moisture content (v/w) achieved was 5, 10, 15, 25 or 30 cm^3^ g^−1^. Treatments were made for each moisture content of each soil type. Each of the IJ concentrations 25, 50 and 100 IJs cm^−2^ was applied to the surface of each sterilized soil type. Pots that served as control were processed in the same ways but without EPN treatments. After 24 h, 10 third-instar larvae were added to the EPN-treated soil in pots for each moisture content and then immediately covered with plastic film, which had tiny or needle-like perforations. A split split plot design with soil texture or humidity level as main factors, nematode concentration as sub-factor, and EPN isolate as sub-sub-factor was implemented. Three replicates for each EPN-treatments to 10 larvae in each soil type at each moisture content were made in pots and then incubated in dark conditions at 25 °C^45,46^. The mortalities of larvae and pupae caused by EPN strains were confirmed under a microscope. The number of emerging fruit fly adults from soil was recorded, and the percentage of cumulative mortality was determined. A split-split-plot design with moisture content as the main factor, nematode concentration as sub-factor, and EPN isolate as sub-sub-factor was applied in data analysis. This experiment was repeated twice.

### Statistical analyses

The larval mortality rates were subjected to ANOVA procedure using the XLSTAT software (version 2016.02.28451, Addinsoft, New York, USA). Datasets were normalized using the Anderson–Darling normality test^47^. Each trial was independently repeated twice. Two-way ANOVA test was performed to examine sources of variation in the observed variables. Significant differences among variables were tested using protected least significant difference and Fisher's protected least significant difference (LSD) test at *P* < 0.01. Differences obtained at levels of *P* < 0.05 were considered significant. Polynomial regression analysis was established to describe the relationship between insect mortality and applied EPN concentration. All multivariate analyses were performed using R software (version 3.4.3, R Core Team^48^ in R Studio, version 1.1.383, RStudio Team^49^).

### Human and animal rights

This article does not contain any studies with human participants or animals performed by any of the authors.
